# ZBP1 inhibits the replication of Senecavirus A by enhancing NF-κB signaling pathway mediated antiviral response in porcine alveolar macrophage 3D4/21 cells

**DOI:** 10.1186/s11658-024-00598-2

**Published:** 2024-05-31

**Authors:** Huizi Li, Tingting Zheng, Ming Chen, Xiaoling Lei, Shuo Li, Xijiao Chen, Shishi Wang, Zhangyong Ning

**Affiliations:** 1 College of Veterinary Medicine, South China Agricultural University, Guangzhou, 510642 China; 2 Maoming Branch, Guangdong Laboratory for Lingnan Modern Agriculture, Maoming, 525000 China

**Keywords:** ZBP1, SVA, NF-κB

## Abstract

**Background:**

Senecavirus A (SVA) caused porcine idiopathic vesicular disease (PIVD) showing worldwide spread with economic losses in swine industry. Although some progress has been made on host factors regulating the replication of SVA, the role of Z-DNA binding protein 1 (ZBP1) remains unclear.

**Methods:**

The expression of ZBP1 in SVA-infected 3D/421 cells was analyzed by quantitative real-time PCR (qRT-PCR) and western blot. Western blot and qRT-PCR were used to detect the effects of over and interference expression of ZBP1 on SVA VP2 gene and protein. Viral growth curves were prepared to measure the viral proliferation. The effect on type I interferons (IFNs), interferon-stimulated genes (ISGs), and pro-inflammatory cytokines in SVA infection was analyzed by qRT-PCR. Western blot was used to analysis the effect of ZBP1 on NF-κB signaling pathway and inhibitor are used to confirm.

**Results:**

ZBP1 is shown to inhibit the replication of SVA by enhancing NF-κB signaling pathway mediated antiviral response. SVA infection significantly up-regulated the expression of ZBP1 in 3D4/21 cells. Infection of cells with overexpression of ZBP1 showed that the replication of SVA was inhibited with the enhanced expression of IFNs (IFN-α, IFN-β), ISGs (ISG15, PKR, and IFIT1) and pro-inflammatory cytokines (IL-6, IL-8, and TNF-α), while, infected-cells with interference expression of ZBP1 showed opposite effects. Further results showed that antiviral effect of ZBP1 is achieved by activation the NF-κB signaling pathway and specific inhibitor of NF-κB also confirmed this.

**Conclusions:**

ZBP1 is an important host antiviral factor in SVA infection and indicates that ZBP1 may be a novel target against SVA.

**Graphical Abstract:**

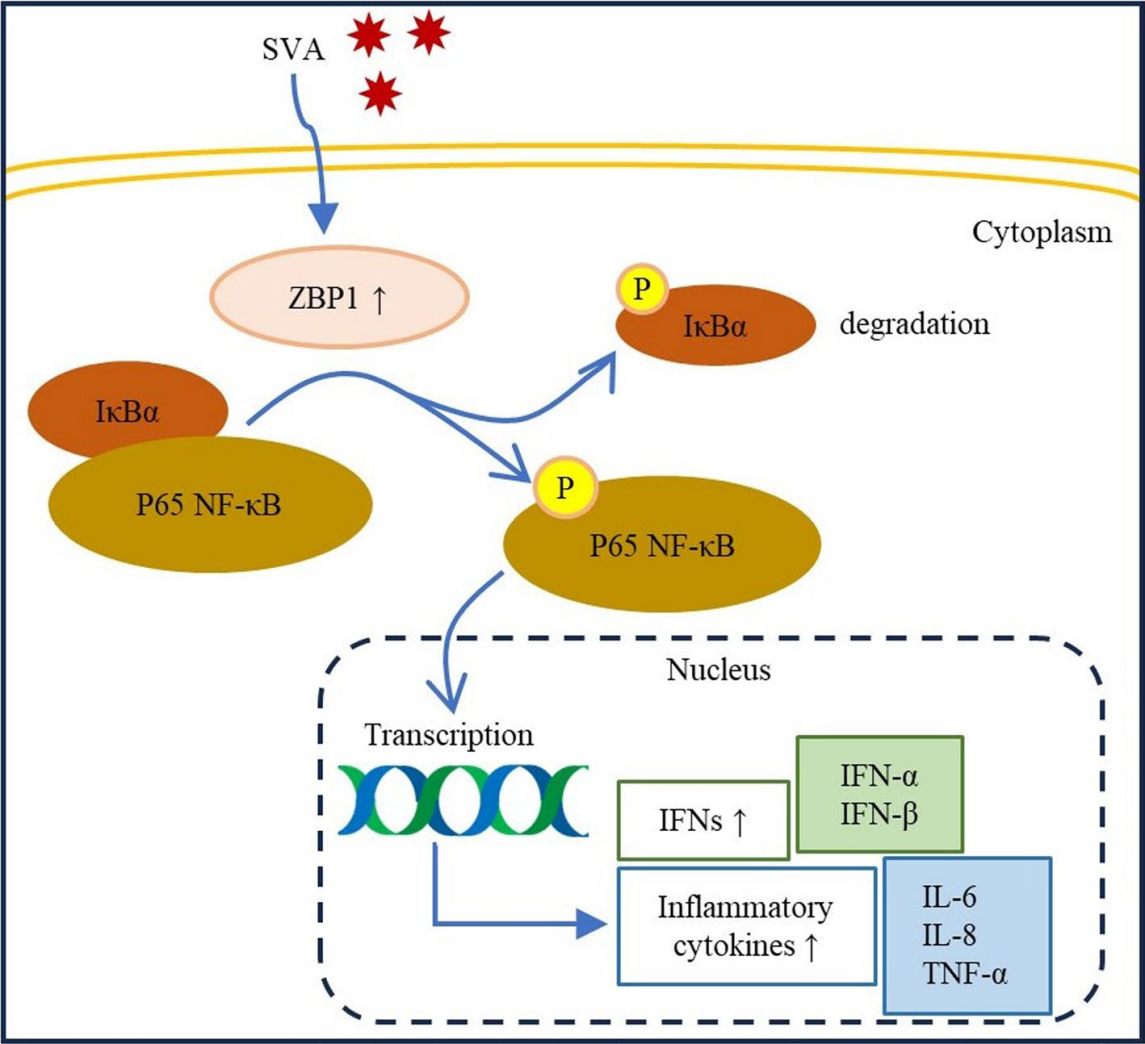

**Supplementary Information:**

The online version contains supplementary material available at 10.1186/s11658-024-00598-2.

## Introduction

Senecavirus A (SVA) belongs to the family *Picornaviridae* and the genus *Senecavirus,* the genome of the virus contains a large single open reading frame that expresses a multiprotein which will be divided into four structural proteins (VP1, VP2, VP3, and VP4) and eight non-structural proteins (L, 2A, 2B, 2C, 3A, 3B, 3C, and 3D) in the infection process of invading cells [1]. SVA induced porcine idiopathic vesicular disease (PIVD) which is characterized by blister-like lesions in the mouth, nose, coronary artery band, and hoof, leading to increased mortality in neonatal pigs [2]. The outbreak of PIVD has been reported in the United States [3], Brazil [4], Colombia [5], Thailand [6], Vietnam [7], Canada [8], and China [9] with economic losses.

Persistent infection of SVA indicated that the virus has the ability to break through the immune barrier of the host by inhibiting the pathway of type I interferons (IFNs) that induce the antiviral responses [10]. Host factors as passive responders to SVA infection will participate in the process of antiviral responses. It has been reported that suppressor of cytokine signaling 1 (SOCS1) facilitates the replication of SVA by inhibiting the type I IFN signaling pathway [11]; IFN-induced protein with tetratricopeptide repeats 3 (IFIT3) targets SVA entry, assembly, and release mediated by a type I IFN antiviral response [12]. Although some progress has been made on host factors regulating the replication of SVA, the role of Z-DNA binding protein 1 (ZBP1) remains unclear.

ZBP1 is one of the left-handed helical Z-conformation nucleic acid (Z-NA) binding protein family members, which contains two N-terminal Z-NA binding regions (Zα1 and Zα2), two intermediate RHIM (RHIM1 and RHIM2), and a C-terminal signaling domain [13, 14]. ZBP1 was recognized as an innate immune sensor that activates innate immune responses, including type I IFN response and NF-κB signaling pathway to effectively resist pathogen invasion [15]. Previous reports showed that ZBP1 restricts murine cytomegalovirus (MCMV) replication by inducing necroptotic apoptosis in host cells [16]; ZBP1 also mediates host cell death preventing replication of herpes simplex virus-1 (HSV-1), varicella-zoster virus (VZV), and vaccinia virus [17–19]; ZBP1 induced RIPK3-RIPK1-caspase-8 dependent apoptosis and RIPK3-MLKL-mediated necroptotic apoptosis in influenza A virus (IAV) infection [20]. While, whether ZBP1 plays a role in SVA infection has not yet been verified.

Here, ZBP1 was reported to inhibit the replication of SVA by enhancing NF-κB signaling pathway mediated antiviral response. These results expand the scope of host factors inhibiting the proliferation of SVA and provide a new potential antiviral target.

## Materials and methods

### Cells, virus, and reagents

The adherent porcine alveolar macrophage (PAM) 3D4/21 cells (American Type Culture Collection: CRL-2843) were cultured at 37 °C and 5% CO_2_ in RPMI 1640 medium (Gibco, USA) supplemented with 10% fetal bovine serum (FBS) (Gibco, USA), 100 IU/mL penicillin, 100 g/mL streptomycin, and 2 mM L-glutamine (Gibco, USA). The SVA strain, CHGDFS-2018, was isolated by our group in 2018 [21]. The heat-inactivated SVA (heat-SVA) was obtained at 70 °C for 2 h in a water bath. Cell counting kit-8 (CCK-8) was purchased from Glpbio (USA), NF-κB inhibitor BAY 11–7082 (99.28%) was obtained from MCE (USA). Anti-NF-κB p65, anti-p-NF-κB p65 (Ser536), anti-IκBα, and anti-p-IκBα (Ser32/36) primary antibodies were purchased from Abmart (China), and anti-β-actin antibody was obtained from Boiss (China). The rabbit anti-VP2 of SVA was stored in our laboratory [11]. The respective horseradish peroxidase (HRP)-conjugated secondary antibodies were purchased from Abcam (Cambridge, UK).

### Overexpression and interference expression of ZBP1

Porcine ZBP1 was amplified from the cDNA of 3D4/21 cells and cloned into the pEGFP-N1 vector in frame with EGFP (Clontech, USA) using the primers designed by Primer 5, F: 5’-CTACTAGCTAGCGGCCACCATGGAGTCGGAGCAGG-3’ and R: 5’-CCCCCCAAGCTTGTGGCCATCTTCT-3’, which contain *Nhe* I and *Hin*d III restriction sites. The constructed plasmids were identified by sequencing and named pEGFP-N1-ZBP1. The specific target sequences of ZBP1, 5’-AATGTCATCATGAGA CACGAG-3’ and negative control (NC): 5’-GCGCTTTCTGCTCAAGAATCT-3’ were designed by Oligoengine, synthesized by Tsingke Biotech (Beijing, China), and cloned into pSuper.neo RNAi plasmid (Oligoengine). The constructed plasmids were identified by sequencing and named pSuper.neo-ZBP1 (shZBP1) and pSuper.neo-NC (shNC). The plasmid was transfected into 3D4/21 cells grown in a 6-well plate using lipofectamine 2000 (Invitrogen, USA) according to the manufacturer’s instructions. After 24 h of transfection, the cells were infected with SVA at an multiplicity of infection (MOI) of 1 for the indicated hours.

### RNA extraction and quantitative real-time PCR (qRT-PCR)

Total cellular RNA was extracted using trizol reagent (Takara, Japan) from 3D4/21 cells and the RNA concentration were determined with a Nano Drop spectrophotometer (Thermo, USA). The RNA was reversely transcribed to cDNA using a reverse transcription kit (Takara, Japan) at a ratio of 500 ng RNA/10 μL of reaction. 100 ng of cDNA per reaction was used to perform qRT-PCR with TB Green Premix Ex Taq Kit (Takara, Japan) in a Light Cycler 480 (Roche, Switzerland). The primers for qRT-PCR were designed using Primer 5 or refer to the previous article, as shown in Table 1 [21]. The relative expressions of the target genes were calculated using the 2^−ΔΔCt^ method with β-actin as an internal control [21].

**Table 1:** Primer sequences for qRT-PCR

Target Gene	Sequence (5′-3′)
VP2	F: TCAACCCACCACCACTTTTA R: CTGCCACCCGCACTTCATTA
IFN-α	F: CTGGCACAAATGAGGAGAAT R: CTCTAGCACTGGCTGGAATGAA
IFN-β	F: CTCTAGCACTGGCTGGAATGAA R: CCGGAGGTAATCTGTAAGTCTGTT
ISG15	F: TGGTGAGGAACGACAAGGGTC R: CCGCAGGCGCAGATTCATAT
PKR	F: AAAGCGGACAAGTCGAAAGG R: TCCACTTCATTTCCATAGTCTTCTGA
IFIT1	F: GGTCTTGGAGGAGATTGAG R: TAACCAGCCTTCTCACCTC
IL-6	F: TGGCTACTGCCTTCCCTACC R: CAGAGATTTTGCCGAGGATGT
IL-8	F: CAGAACTTCGATGCCAGTG R: GGTCCAGGCAGACCTCTTT
TNF-α	F: CGCATCGCCGTCTCCTACCA R: TGCCCAGATTCAGCAAAGTCCAG

### Preparation of anti-porcine ZBP1 IgG

The sequence of ZBP1 was designed by Primer 5 and inserted into the pET-32a ( +) vector to construct the His-ZBP1 plasmid using the primers that F: 5’-CCCCCCCAAGCTTGCATGGAGTCGGAGCAGGCCA-3’ and R: 5’-CCGCCGCTCGAGTTAGTGGCCATCTTCTGTGGTTC-3’ containing *Hin*d III and *Xho* I restriction sites. Expression of the His-ZBP1 fusion protein was induced by isopropyl β-D-1-thiogalactopyranoside (IPTG). Purification of the ZBP1 protein, antiserum preparation, and polyclonal rabbit anti-porcine ZBP1 IgG purification were carried out according to our previous report [22]. After immunization, the titers of the rabbit antisera were determined by enzyme linked immunosorbent assay (ELISA) [22].

### Western blot

Western blot was performed according to our previous research [11]. Briefly, cellular proteins were extracted from different samples and separated with 10% sodium dodecyl sulfate polyacrylamide gel electrophoresis (SDS-PAGE) and transferred onto a nitrocellulose membrane (Pall, USA). After washed with tris-buffered saline containing tween-20 (TBST), the membrane was blocked with 5% (w/v) skim milk, then, incubated with the corresponding primary and secondary antibodies. Finally, the enhanced chemiluminescence detection reagent (Genview, USA) was used to detect the protein, and the grayscale was analyzed by Image J software.

### Preparation of viral growth curves

Cell suspensions were collected at 6, 12, 24, 36, and 48 h post infection (h.p.i), the 50% tissue culture infective dose (TCID_50_) was performed in 3D4/21 cells and calculated using the Reed-Muench method [11]. In brief, 3D4/21 cells were cultured in 96-well cell culture plates and seeded with 100 μL viral solution in a tenfold serial dilution (from 10^–1^ to 10^–8^), after 2 h of adsorption, cells were washed twice with 1640 medium, then, cultured in 1640 medium supplemented with 2% FBS, cell pathogenic effects (CPE) were determined after 7 days.

### Cytotoxicity assay and inhibitor assays

BAY 11–7082 is dissolved in 0.1% (v/v) DMSO and the cytotoxic of BAY 11–7082 in 3D4/21 cells was detected by CCK-8 reagent according to previous report [11]. 3D4/21 cells were cultured in 96-well plates and treated with BAY 11–7082 (0.5, 1, 2, and 5 μM). After 24 h, 10 μL of CCK-8 solution was added and cell viability was determined by a microplate reader (Thermo, USA).

In NF-κB inhibitor assays, 3D4/21 cells were divided into three groups: cells were treated with 0.1% (v/v) DMSO for 16 h and infected with SVA (MOI of 1) for 24 h was used as a control (SVA + DMSO); cells were transfected with pEGFP-N1-ZBP1, treated with 0.1% (v/v) DMSO for 16 h, and infected with SVA (MOI of 1) for 24 h (ZBP1 + SVA + DMSO); cells were transfected with pEGFP-N1-ZBP1, treated with BAY 11–7082 (5 μM) for 16 h, and infected with SVA (MOI of 1) for 24 h (ZBP1 + SVA + BAY 11–7082).

### Statistical analysis

Results were analyzed using GraphPad Prism version 8.0 software (GraphPad Software, USA) and the results of statistical analysis were reported as means ± SD. Comparisons between the groups were performed using Student’s *t*-test. A value of *P* < 0.05 was considered statistically significant. (^*^P < 0.05, ^**^P < 0.01, ns, not significant).

## Results

### SVA infection up-regulates ZBP1 expression in 3D4/21 cells

**Fig. 1:**
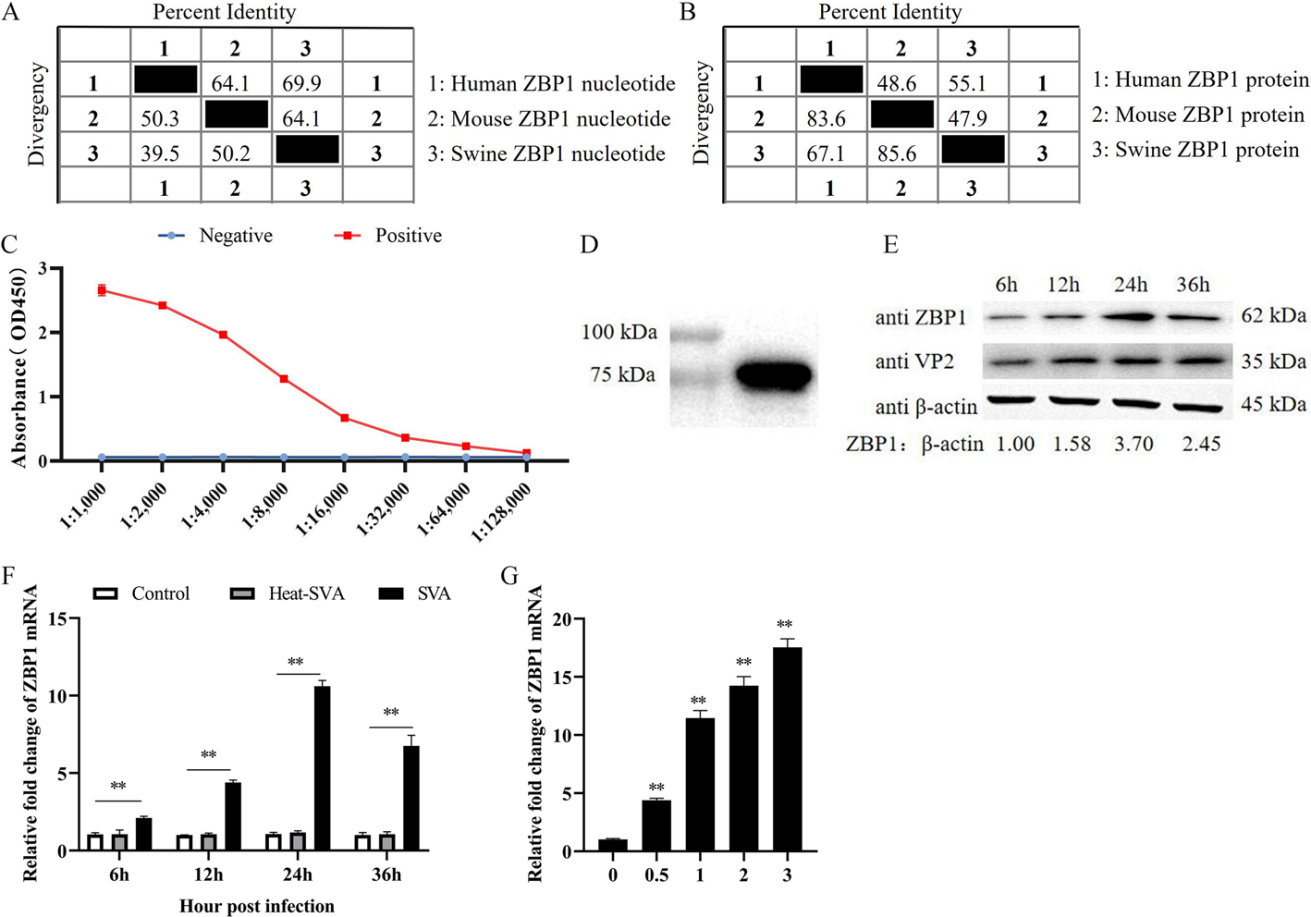
SVA infection up-regulates ZBP1 expression in 3D4/21 cells. **A**, **B** The homology of ZBP1 nucleotide and protein between swine, human, and mouse were aligned. **C** The titers of serum were detected by ELISA, positive: immune serum, negative: unimmunized serum. **D** The recombinant His-ZBP1 protein were detected by western blotting using prepared rabbit polyclonal anti-porcine ZBP1 IgG. **E** 3D4/21 cells were infected with SVA, protein levels of ZBP1 and VP2 were detected by western blotting. **F** 3D4/21 cells were infected with SVA and heat-SVA at an MOI of 1, mock-infected served as a control. Cells were harvested at 6, 12, 24 and 36 h.p.i, the mRNA expression levels of ZBP1 were measured by qRT-PCR. **G** The mRNA expression levels of ZBP1 were measured by qRT-PCR in 3D4/21 cells infected with SVA at MOIs of 0.5, 1, 2, and 3 for 24 h. All experiments were repeated three times independently. ^*^P < 0.05, ^**^P < 0.01

Commercial anti-ZBP1 antibodies are only available for humans, while, the homology is only 69.9% of nucleotide sequences between humans and swine ZBP1 (Fig. 1A), and is 55.1% of amino acid (Fig. 1B). To ensure protein detection in following experiments, anti-porcine ZBP1 IgG was prepared. ELISA showed that rabbit anti-porcine ZBP1 serum has excellent specificity until dilutions reach 1:16,000 (Fig. 1C). The prepared anti-porcine ZBP1 IgG was used as the primary antibody to detect the recombinant His-ZBP1 by western blotting. The results show that the prepared IgG has favorable specificity (Fig. 1D).

3D4/21 cells were infected by SVA (MOI of 1), western blot showed that SVA infection promoting the expression of ZBP1 from 12 to 36 h.p.i (Fig. 1E). 3D4/21 cells were infected with SVA (MOI of 1) or heat-SVA, and mock-infected served as a control. The expression levels of ZBP1 mRNA were measured by qRT-PCR at 6, 12, 24, and 36 h.p.i. The results showed that the mRNA of ZBP1 was significantly up-regulated from 6 to 36 h.p.i (*P* < 0.01) (Fig. 1F). While, the heat-SVA could not induce changes expression levels of ZBP1, indicating that the expression of ZBP1 depends on SVA replication. 3D4/21 cells were infected with SVA at different dose (MOIs of 0.5, 1, 2, and 3) for 24 h and the expression of ZBP1 was induced by SVA infection (*P* < 0.01) (Fig. 1G). These results indicate that SVA significantly up-regulates the expression of ZBP1 in 3D4/21 cells.

### ZBP1 inhibits the replication of SVA

**Fig. 2:**
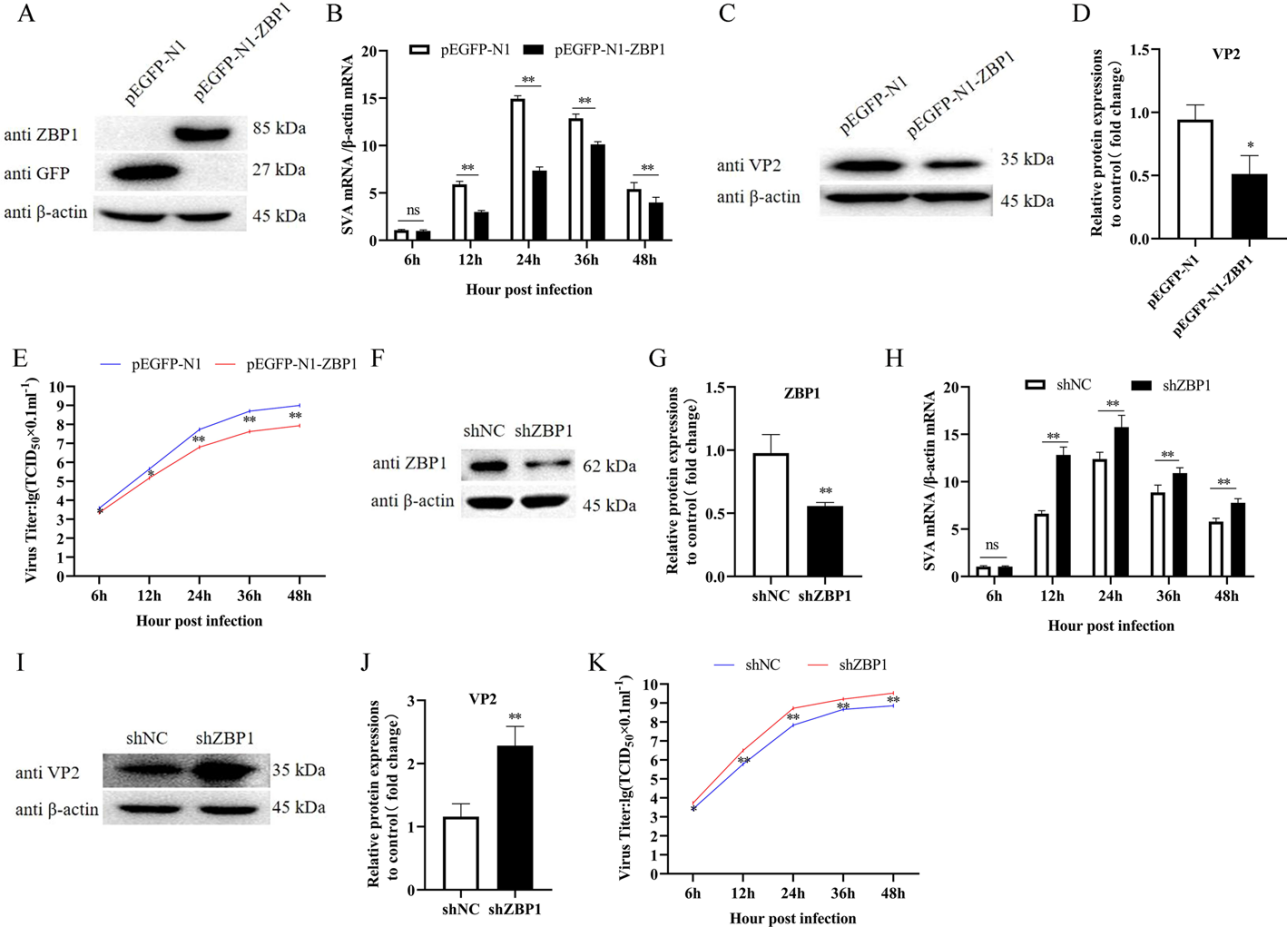
ZBP1 inhibits the replication of SVA. **A** The expression levels of ZBP1 were detected by western blotting in 3D4/21 cells transfected with pEGFP-N1 or pEGFP-N1-ZBP1 for 24 h. **B** The mRNA expression of SVA VP2 gene was analyzed by qRT-PCR at 6, 12, 24, 36, and 48 h.p.i in SVA (MOI of 1) infected 3D4/21 cells after transfected with pEGFP-N1 or pEGFP-N1-ZBP1 for 24 h. **C**, **D** The expression levels of VP2 protein were detected by western blotting at 24 h.p.i and quantitatively analyzed by Image J. **E** Virus titers were determined in 3D4/21 cells using the Reed-Muench method. **F**, **G** The expression of ZBP1 was detected by western blotting in 3D4/21 cells transfected with shNC or shZBP1 for 24 h and analyzed by Image J. **H** The expression of SVA VP2 gene were analyzed by qRT-PCR in SVA (MOI of 1) infected 3D4/21 cells after transfected with shNC or shZBP1 for 24 h. **I**, **J** The expression levels of VP2 protein were detected by western blotting at 24 h.p.i and quantitatively analyzed by Image J. **K** Virus titers were determined in 3D4/21 cells. All experiments were repeated three times independently. ^*^P < 0.05, ^**^P < 0.01, ns: not significant

To investigate the role of ZBP1 on SVA proliferation, 3D4/21 cells with ZBP1-overexpression and -interference expression were used for SVA infection. In ZBP1-overexpression cells, pEGFP-N1 or pEGFP-N1-ZBP1 was transfected into 3D4/21 cells, respectively (Fig. 2A). The results of qRT-PCR showed that ZBP1 inhibited the copy number of SVA VP2 gene from 12 to 48 h.p.i compared with the control (*P* < 0.01) (Fig. 2B). Western blots showed that ZBP1 overexpression significantly inhibited the expression of SVA VP2 protein (*P* < 0.05) (Fig. 2C, D). Transfection of pEGFP-N1-ZBP1 resulted in a significant decrease in viral titers of approximately three- to tenfold at 6 (*P* < 0.05), 12 (P < 0.05), 24 (*P* < 0.01), 36 (*P* < 0.01), and 48 h.p.i (P < 0.01) compared to the control (Fig. 2E). In ZBP1-interference expression cells, shZBP1 and shNC was transfected into 3D4/21 cells, respectively, and the expression of ZBP1 was only 0.5 in cells with ZBP1-interference expression (Fig. 2F, G). The mRNA levels of SVA VP2 gene was significantly higher in ZBP1-interference group from 12 to 48 h.p.i compared to the control (*P* < 0.01) (Fig. 2H). The results of western blot revealed that VP2 protein expression were also raised in cells with impeded ZBP1 expression (*P* < 0.01) (Fig. 2I, J). Virus titers in ZBP1-interference cells were significantly higher at 6 (*P* < 0.05), 12 (*P* < 0.01), 24 (*P* < 0.01), 36 (*P* < 0.01), and 48 h.p.i (*P* < 0.01) compared to the control, and approximately tenfold increase at 24 h.p.i (Fig. 2K). These results show that ZBP1 inhibits the replication of SVA.

### ZBP1 promotes the expression of IFNs, ISGs, and pro-inflammatory cytokines in SVA infection

**Fig. 3:**
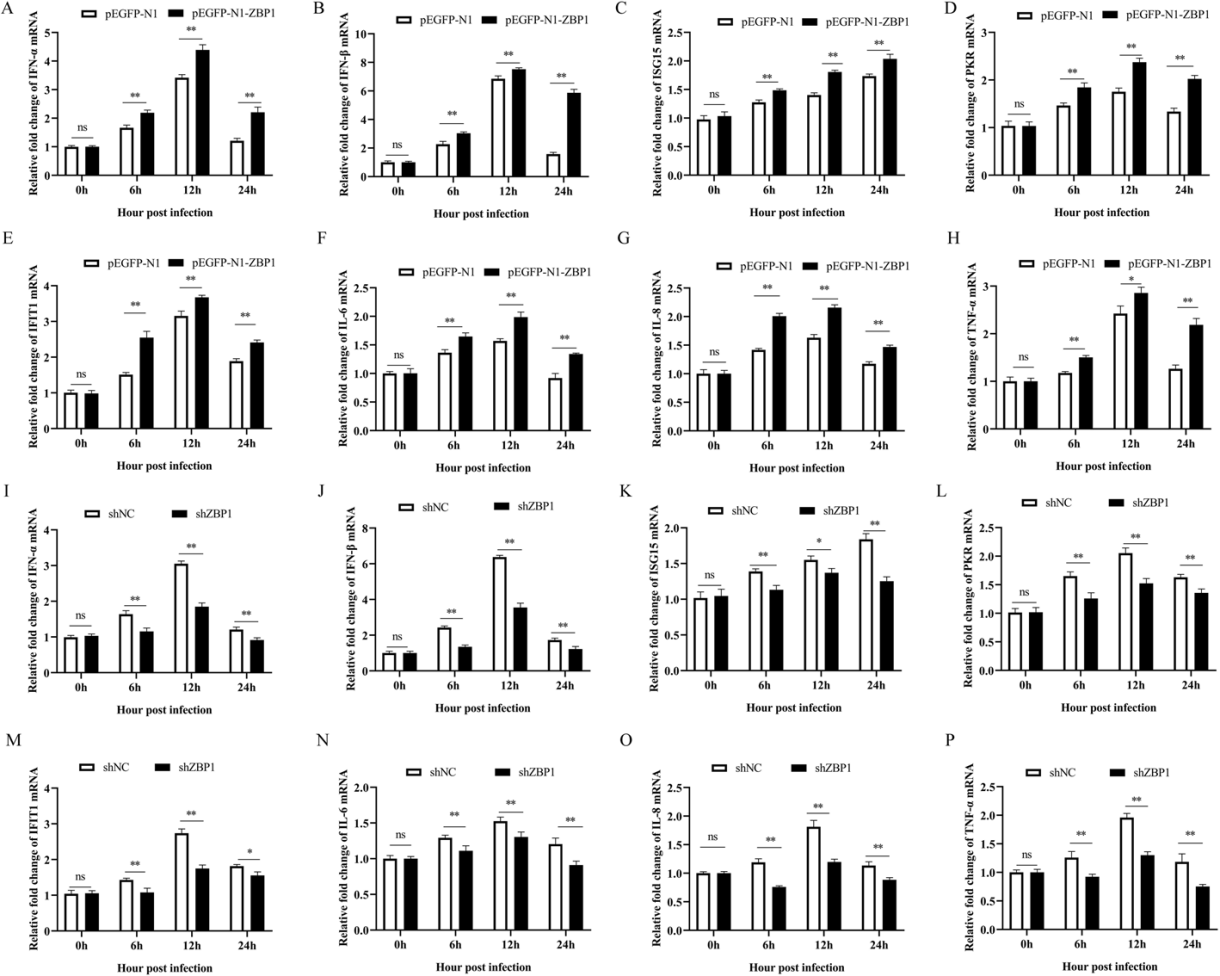
ZBP1 promotes the expression of IFNs, ISGs, and pro-inflammatory cytokines in SVA infection. **A**–**H** SVA (MOI of 1) infected 3D4/21 cells after transfected with pEGFP-N1 or pEGFP-N1-ZBP1 for 24 h, the mRNA expression of IFN-α, IFN-β, PKR, ISG15, IFIT1, IL-6, IL-8, and TNF-α were detected by qRT-PCR at 0, 6, 12, and 24 h.p.i. **I**–**P** SVA (MOI of 1) infected 3D4/21 cells after transfected with shNC or shZBP1 for 24 h, the mRNA expression of IFN-α, IFN-β, PKR, ISG15, IFIT1, IL-6, IL-8, and TNF-α were detected by qRT-PCR at 0, 6, 12, and 24 h.p.i. All experiments were repeated three times independently. ^*^P < 0.05, ^**^P < 0.01, ns: not significant

3D4/21cells with ZBP1-overexpression and -interference expression were used to determine whether ZBP1 had an effect on the expression of type I IFNs and pro-inflammatory cytokines in SVA infection. In ZBP1-overexpression cells, the mRNA expression of IFN-α, IFN-β, PKR, ISG15, IFIT1, IL-6, and IL-8 were significantly higher at 6, 12, and 24 h.p.i (*P* < 0.01), TNF-α was significantly higher at 6 (*P* < 0.01), 12 (*P* < 0.05), and 24 h.p.i (*P* < 0.01) compared to the control (Fig. 3A–H). While, the opposite results were shown in ZBP1-interference expression cells. Impeded ZBP1 expression decreased the expression of IFN-α, IFN-β, PKR, IL-6, IL-8 and TNF-α from 6 to 24 h.p.i (*P* < 0.01), ISG15 at 6 (*P* < 0.01), 12 (*P* < 0.05), and 24 (*P* < 0.01) h.p.i, IFIT1 at 6 (*P* < 0.01), 12 (*P* < 0.01), and 24 (*P* < 0.05) h.p.i, compared to the control (Fig. 3I–P). These results indicate that ZBP1 promotes the expression of type I IFNs, ISGs, and pro-inflammatory cytokines in SVA infection.

### ZBP1 activates NF-κB signaling pathway in SVA-infected cells

**Fig. 4:**
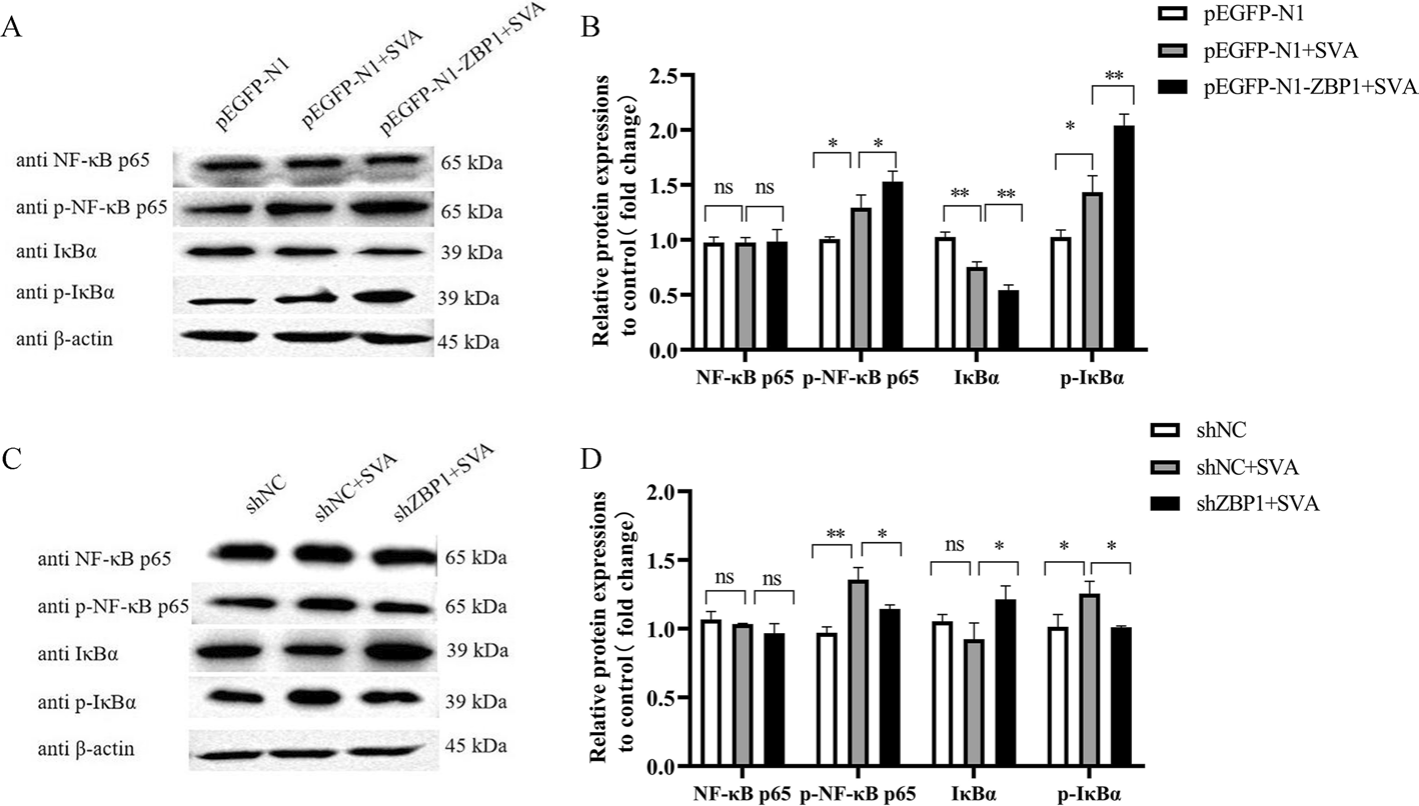
ZBP1 activates NF-κB signaling pathway in SVA-infected cells. **A**, **B** SVA (MOI of 1) infected or unifected 3D4/21 cells after transfected with pEGFP-N1 or pEGFP-N1-ZBP1 for 24 h, the expression levels of NF-κB p65, p-NF-κB p65, IκBα, and p-IκBα protein were detected by western blotting and quantitatively analyzed by Image J. **C**, **D** SVA (MOI of 1) infected or unifected 3D4/21 cells after transfected with shNC or shZBP1 for 24 h, the expression levels of NF-κB p65, p-NF-κB p65, IκBα, and p-IκBα protein were detected by western blot and quantitatively analyzed by Image J. All experiments were repeated three times independently. ^*^P < 0.05, ^**^P < 0.01, ns: not significant

NF-κB is a key regulator of IFN activation and inflammatory cytokine production [23]. To verify whether inhibitory effect of ZBP1 on SVA replication is approached via NF-κB signaling pathway, infection experiments was carried out in 3D4/21 cells with ZBP1-over and -interference expression. In overexpression group, SVA infection increased the expression of p-NF-κB p65 (*P* < 0.05) and p-IκBα (*P* < 0.05), and decreased the expression of IκBα (*P* < 0.01), compared to pEGFP-N1 group; ZBP1-overexpression increased the expression of p-NF-κB p65 (*P* < 0.05) and p-IκBα (*P* < 0.01), and decreased the expression of IκBα (*P* < 0.01), compared to pEGFP-N1 + SVA, no significant difference in the protein levels of NF-κB at 24 h.p.i (Fig. 4A, B). In interference expression group, SVA infection increased the expression of p-NF-κB p65 (*P* < 0.01) and p-IκBα (*P* < 0.05), compared to shNC; impeded ZBP1 expression decreased the expression of p-NF-κB p65 (*P* < 0.05) and p-IκBα (*P* < 0.05), and increased the expression of IκBα (*P* < 0.05), compared to shNC + SVA, no significant difference in the protein levels of NF-κB at 24 h.p.i (Fig. 4C, D). These results indicate that ZBP1 activates NF-κB signaling pathway in SVA-infected cells.

### ZBP1 activating NF-κB signaling pathway was verified by the specific inhibitor

**Fig. 5:**
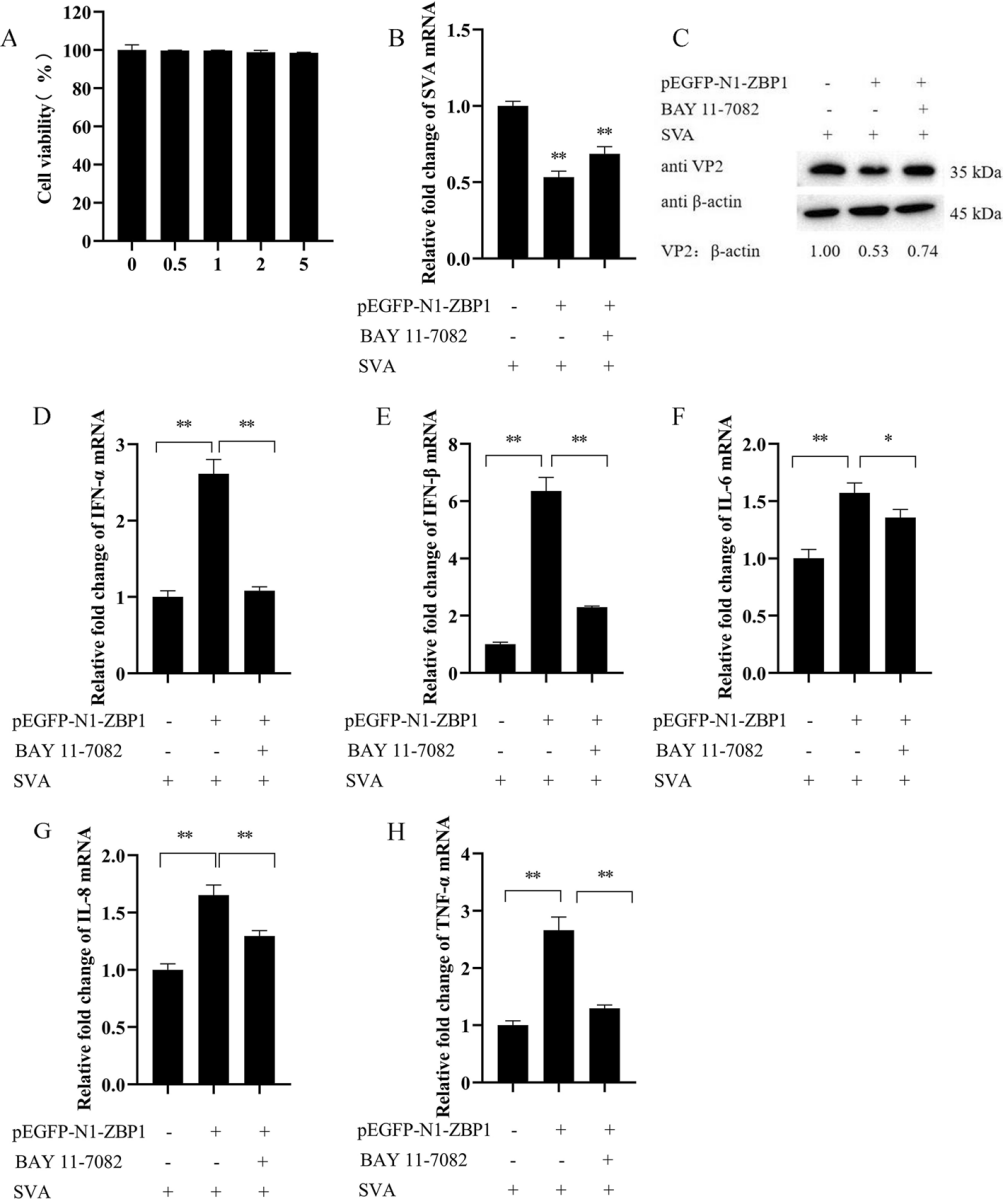
ZBP1 activating NF-κB signaling pathway was verified by the specific inhibitor. **A** The cytotoxic effect of BAY 11–7082 in 3D4/21 cells was determined by CCK-8 assay. **B **3D4/21 cells were transfected with pEGFP-N1 or pEGFP-N1-ZBP1, and pre- treated with DMSO or BAY 11–7082 (5 μM) for 16 h, then, infected with SVA at an MOI of 1 for 24 h. The expression of SVA VP2 gene was analyzed by qRT-PCR. **C** The expression levels of VP2 protein were detected by western blot and quantitatively analyzed by Image J. **D**–**H** The expression of IFN-α, IFN-β, IL-6, IL-8, and TNF-α were detected by qRT-PCR. All experiments were repeated three times independently. ^*^P < 0.05, ^**^P < 0.01

An NF-κB-specific inhibitor, BAY 11–7082, was used to confirm the activation of ZBP1 in NF-κB signaling pathway in SVA infection. In cytotoxicity assays, 3D4/21 cells were treated with BAY 11–7082 at concentrations of 0.5, 1, 2, and 5 μM for 24 h. The results showed that BAY 11–7082 below 5 μM had no obvious effect of cell viability (Fig. 5A). Then, 3D4/21 cells were transfected with pEGFP-N1-ZBP1 and incubated by inhibitor (5 μM) for 16 h. The qRT-PCR showed that ZBP1 inhibited the mRNA expression of the SVA VP2 gene (*P* < 0.01), while, when BAY 11–7082 was present, the inhibition effect of ZBP1 on SVA was reduced (Fig. 5B). The results of western blot showed that the VP2 protein levels were increased in BAY 11–7082 group compared with the ZBP1-SVA-infected group, which was consistent with the mRNA expression levels of SVA VP2 gene (Fig. 5C). In ZBP1-SVA-infected group, ZBP1 significantly increased IFN-α, IFN-β, IL-6, IL-8 and TNF-α (*P* < 0.01) compared to SVA-infected cells, while this facilitation was attenuated when the inhibitor was present, the expression of IFN-α (*P* < 0.01), IFN-β (*P* < 0.01), IL-6 (*P* < 0.05), IL-8 (*P* < 0.01) and TNF-α (*P* < 0.01) were decreased compared to the ZBP1-SVA-infected group (Fig. 5D–H). These results show that the inhibitory effect of ZBP1 on SVA replication is dependent on NF-κB.

## Discussion

The persist infection of SVA has led to the rapid spread of PIVD in swine farming countries worldwide [10]. Also, SVA infection has been reported in the major producing areas of China since the first outbreak in Guangdong province in 2015 [9]. An undeniable fact is that there are still no commercial vaccines and therapeutic agents for SVA and this makes it more necessary to conduct in-depth research. Host factors play a role in viral infection and may be targets against the virus. Here, ZBP1 was reported to inhibit the replication of SVA, suggesting that it is a host antiviral protein.

SVA establishes persistent infection by escaping host immunity, making disease control difficult [10]. NF-κB signaling pathway plays a key role in mediating inflammation, immune response to pathogen infection, proliferation, apoptosis, and other cellular activities [24]. In this research, ZBP1-mediated NF-κB signaling by promoting the phosphorylation of NF-κB p65 and IκBα, increasing the production of downstream IFNs and pro-inflammatory factors, and specific inhibitor of NF-κB also proved this. Some reports have shown that ZBP1 regulates the inflammatory response in viral infection, which is consistent with our results [25, 26]. SVA has multiple strategies to evade host immunity, our previous report showed that SOCS1 is facilitated by SVA to inhibit NF-κB mediated antiviral responses [11], to explore whether there is a link between these two proteins, we verified the expression levels of SOCS1 protein in cells with overexpression of ZBP1, and the results showed that ZBP1 inhibited SVA replication independently of SOCS1 (Additional file 1: Supplementary Fig. 1). Our results demonstrate that ZBP1 positively regulates type I IFN signaling and promotes the expression of IFNs and pro-inflammatory cytokines through NF-κB signaling pathway, which provides new insights in the regulation of ZBP1 on innate immune.

Type I IFNs, ISGs, pro-inflammatory cytokines, and chemokines participate in innate immunity and stimulate adaptive immunity, IFNs is a key immune molecule to against SVA infection [12, 27, 28]. ZBP1 promotes the production of these antiviral factors and acts as a sensor of innate immunity to exert its antiviral protein function in SVA-infected cells, while, there was no obvious regularity in the expression of ZBP1 and SVA VP2 protein (Additional file 2: Supplementary Fig. 2). As an ISG, ZBP1 promotes the expression of type I IFNs, which is the reason of overexpression or interference with ZBP1 can affect the production of IFNs [20]. As a nucleic acid sensor, the Zα domain of ZBP1 triggers inflammatory response by binding to Z-conformational nucleic acids (Z-DNA/Z-RNA) [29], however, whether SVA can form Z-RNA during replication needs to be further tested. In addition, the expression of ZBP1 is upregulated in the infection of a variety of viruses, such as influenza virus [30], coxsackievirus A6 [31], and HSV-1 [17]. ZBP1 is also considered to be a limiting factor of HSV-1, indicating that ZBP1 is an important antiviral molecule.

## Conclusions

This report represents that ZBP1 inhibits SVA replication by activating NF-κB and increasing the expression of type I IFNs as well as related inflammatory factors. These findings provide a new insight on host factors restricting the replication of SVA and indicates that ZBP1 may be a novel target against the virus.

### Supplementary Information

Additional file 1: Supplementary Fig. 1 The expression levels of SOCS1 in cells with overexpression of ZBP1.

Additional file 2: Supplementary Fig. 2 Relationship between the expression levels of ZBP1 and SVA VP2 proteins. **A** The expression of pEGFP-N1-ZBP1, SVA VP2, and β-actin protein in SVA-infected 3D4/21 cells with overexpression of pEGFP-N1-ZBP1. **B** The association between the expression levels of pEGFP-N1-ZBP1 and VP2 protein in (**A**) was quantitatively analyzed by image J. (C) The expression of ZBP1, SVA VP2, and β-actin protein in SVA-infected 3D4/21 cells with interference expression of ZBP1. (D) The association between the expression levels of ZBP1 and VP2 protein in (C) was quantitatively analyzed by image J. (E) The association between the expression levels of ZBP1 and VP2 protein in SVA infected 3D4/21 cells in Fig. 1E was quantitatively analyzed by image J.

Additional file 3.

## Data Availability

All data generated or analysed during this study are included in this published article.

## Abbreviations

CCK-8: Cell counting kit-8
CPE: Cell pathogenic effects
HSV-1: Herpes simplex virus-1
IAV: Influenza A virus
IFIT3: IFN-induced protein with tetratricopeptide repeats 3
IFNs: Interferons
ISGs: Interferon-stimulated genes
IPTG: Isopropyl β-D-1-thiogalactopyranoside
MCMV: Murine cytomegalovirus
PIVD: Porcine idiopathic vesicular disease
qRT-PCR: Quantitative real-time PCR
SDS-PAGE: Sodium dodecyl sulfate polyacrylamide gel electrophoresis
shNC: PSuper.neo-NC
shZBP1: PSuper.neo-ZBP1
SOCS1: Suppressor of cytokine signaling1
SVA: Senecavirus A
TCID_50_: 50% Tissue culture infective dose
VZV: Varicella-zoster virus
ZBP1: Z-DNA binding protein 1
Zα: N-terminal Z-NA binding regions
