# Supplementary figures and images for: ZBP1 inhibits the replication of Senecavirus A by enhancing NF-κB signaling pathway mediated antiviral response in porcine alveolar macrophage 3D4/21 cells
Source: Cell Mol Biol Lett. 2024 May 31;29:83. doi: 10.1186/s11658-024-00598-2 (PMC11140869; doi:10.1186/s11658-024-00598-2)

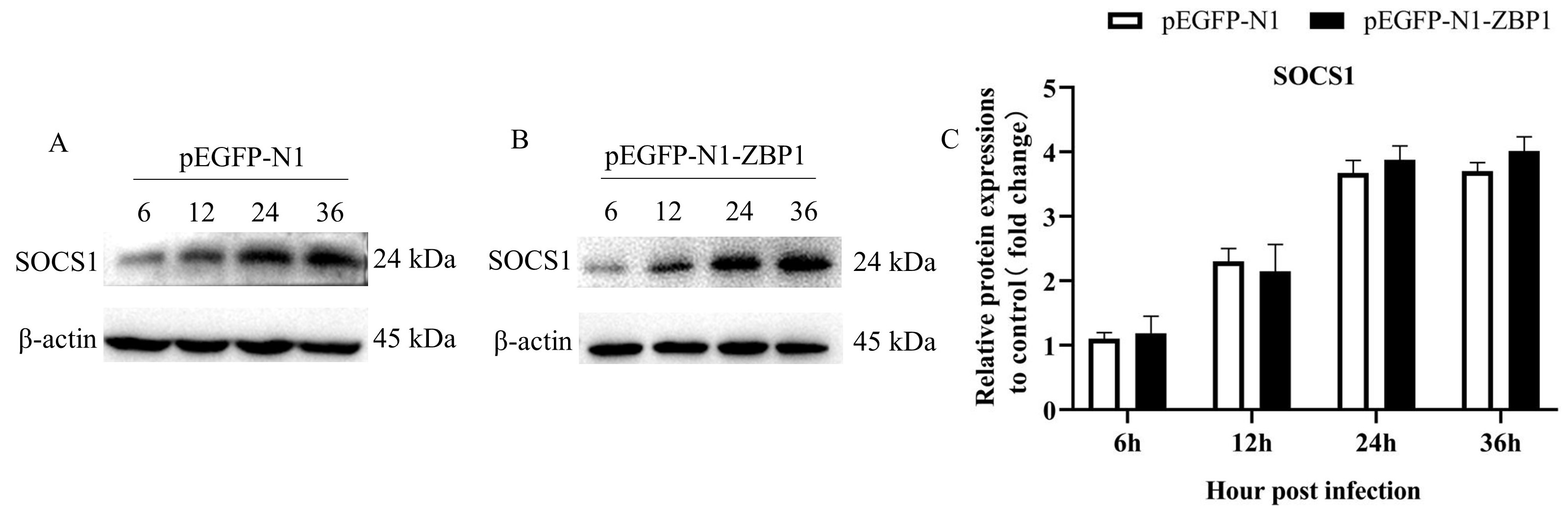

Supplement: Supplementary file 1 — Additional file 1: Supplementary Fig. 1 The expression levels of SOCS1 in cells with overexpression of ZBP1. [file 11658_2024_598_MOESM1_ESM.jpg]

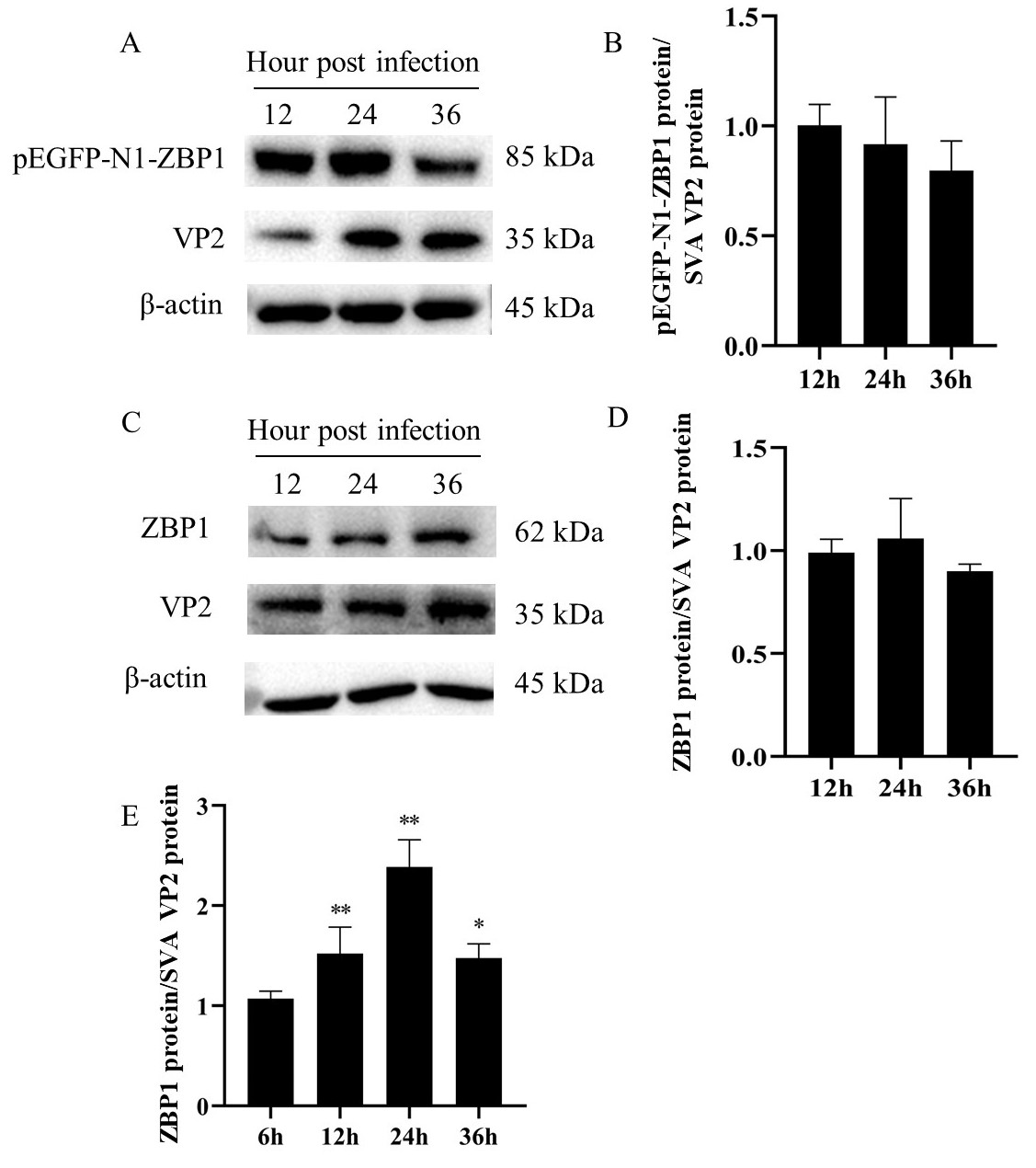

Supplement: Supplementary file 2 — Additional file 2: Supplementary Fig. 2 Relationship between the expression levels of ZBP1 and SVA VP2 proteins. A The expression of pEGFP-N1-ZBP1, SVA VP2, and β-actin protein in SVA-infected 3D4/21 cells with overexpression of pEGFP-N1-ZBP1. B The association between the expression levels of pEGFP-N1-ZBP1 and VP2 protein in (A) was quantitatively analyzed by image J. (C) The expression of ZBP1, SVA VP2, and β-actin protein in SVA-infected 3D4/21 cells with interference expression of ZBP1. (D) The association between the expression levels of ZBP1 and VP2 protein in (C) was quantitatively analyzed by image J. (E) The association between the expression levels of ZBP1 and VP2 protein in SVA infected 3D4/21 cells in Fig. 1E was quantitatively analyzed by image J. [file 11658_2024_598_MOESM2_ESM.jpg]
