# Supplementary material for: ZBP1 inhibits the replication of Senecavirus A by enhancing NF-κB signaling pathway mediated antiviral response in porcine alveolar macrophage 3D4/21 cells
Source: Cell Mol Biol Lett. 2024 May 31;29:83. doi: 10.1186/s11658-024-00598-2 (PMC11140869; doi:10.1186/s11658-024-00598-2)
Supplement: Supplementary file 3 — Additional file 3. [file 11658_2024_598_MOESM3_ESM.pdf]

Supplementary data

Fig.1 SVA infection up-regulated ZBP1 expression in 3D4/21 cells.

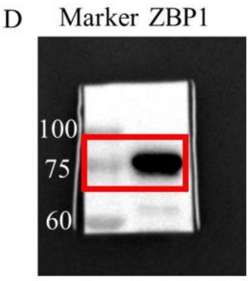

Raw image of western blots for ZBP1 recombinant protein (D).

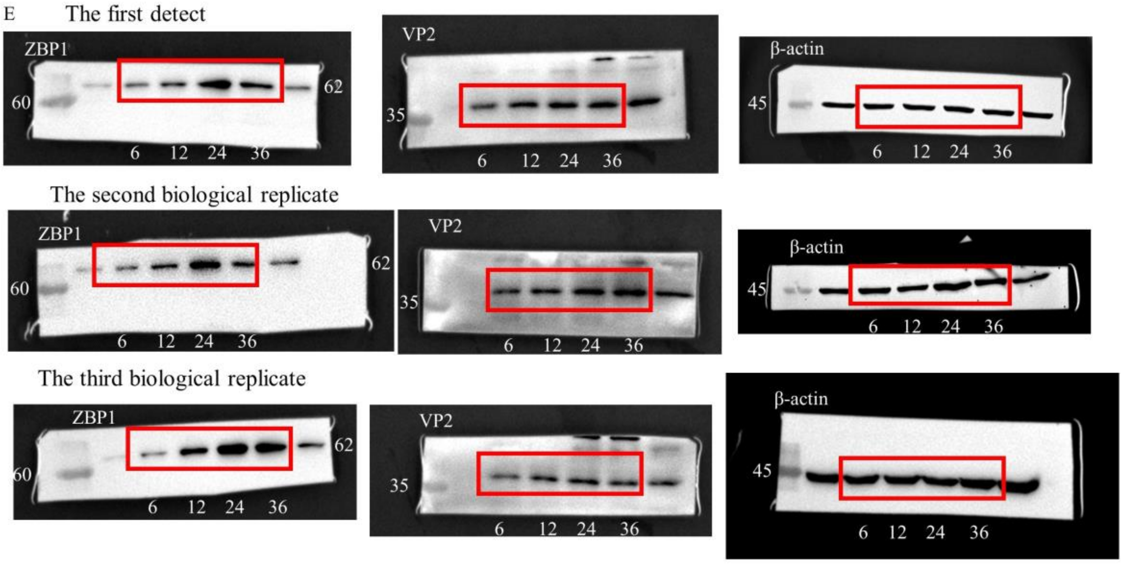

Raw image of western blot for ZBP1, VP2, and β-actin (E).

Fig.2 ZBP1 inhibits the replication of SVA.

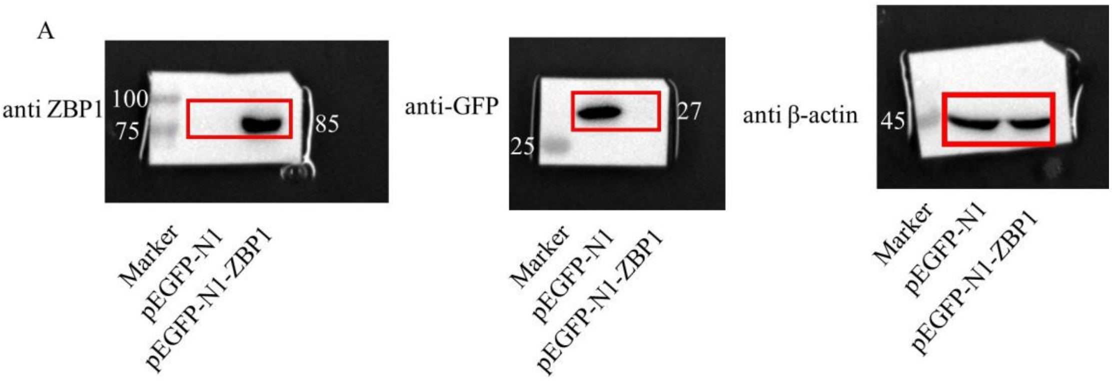

Raw image of western blot for pEGFP-N1-ZBP1, pEGFP-N1, and β-actin (A).

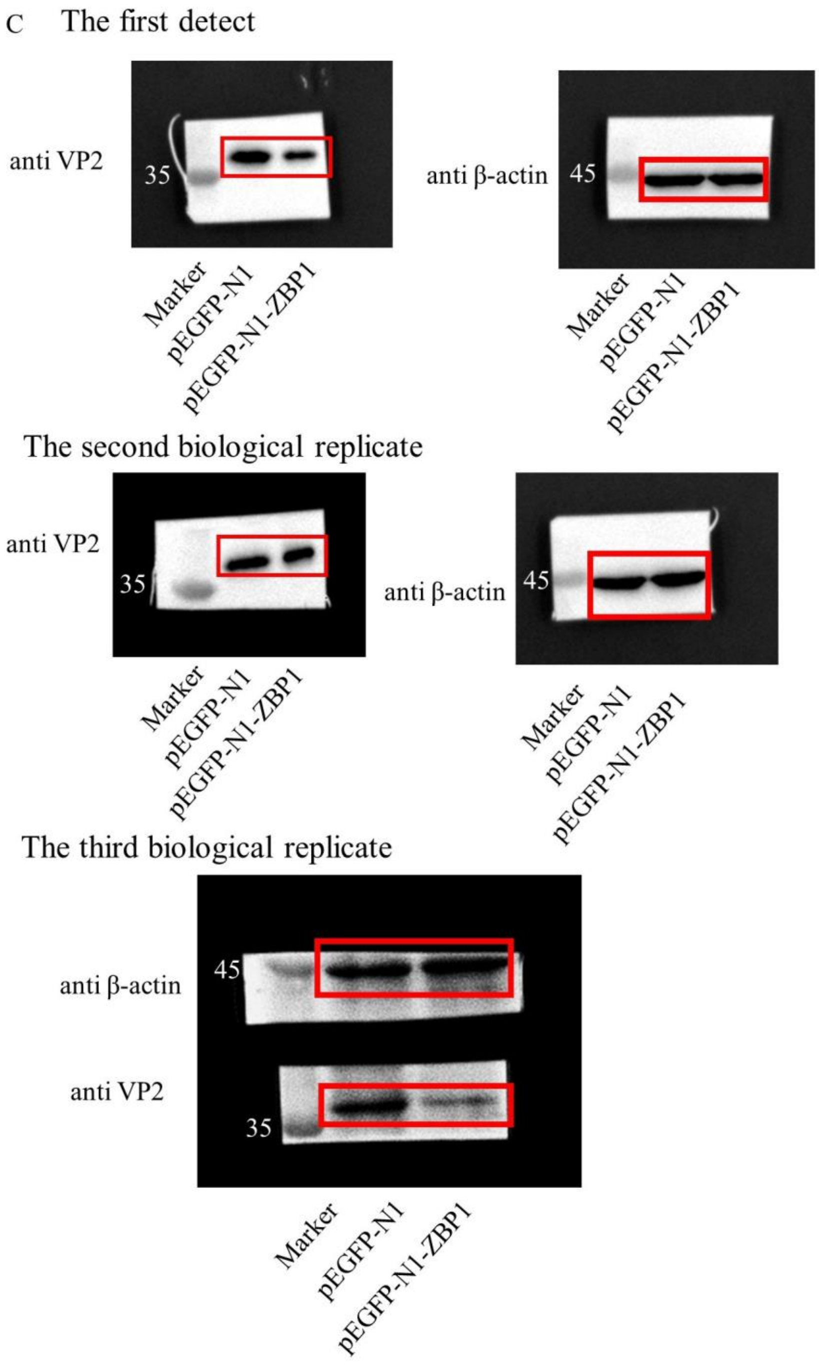

Raw image of western blot for VP2 and β-actin (C).

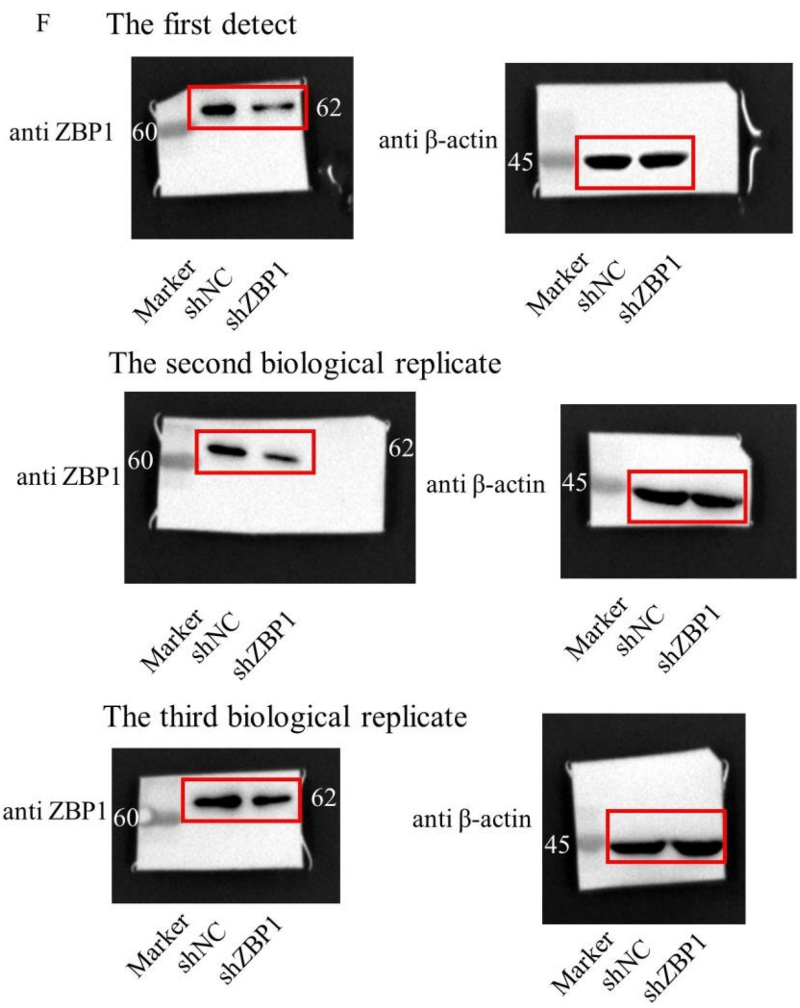

Raw image of western blot for ZBP1 and β-actin (F).

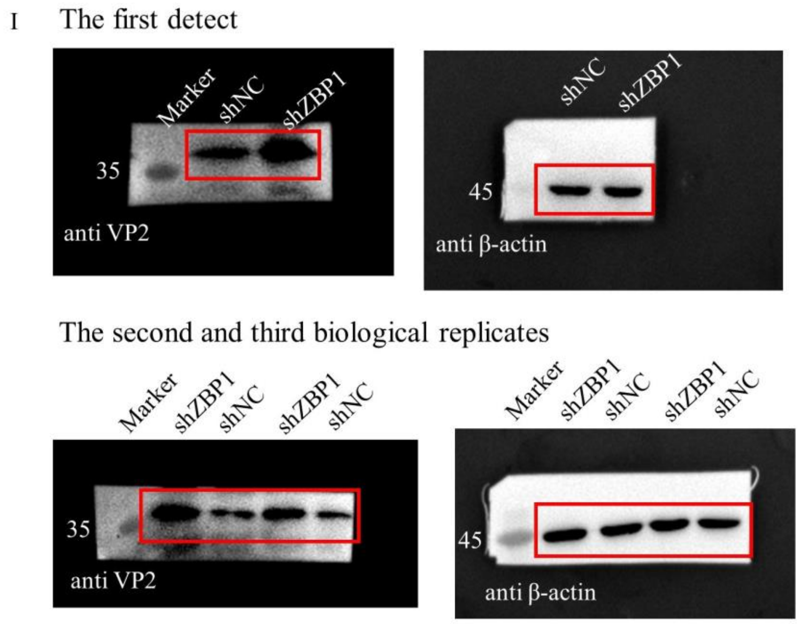

Raw image of western blot for VP2 and β-actin (I).

Fig.4 ZBP1 activates NF-κB signaling pathway in SVA infected cells.

A The first detect

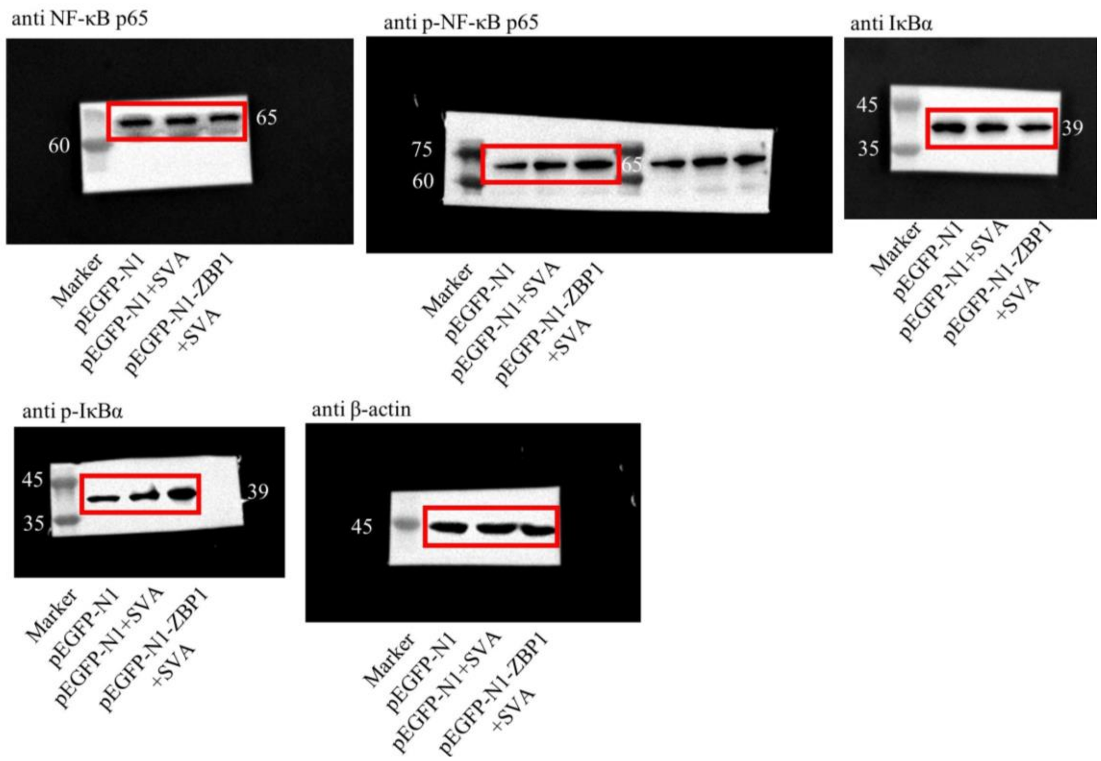

The second biological replicate

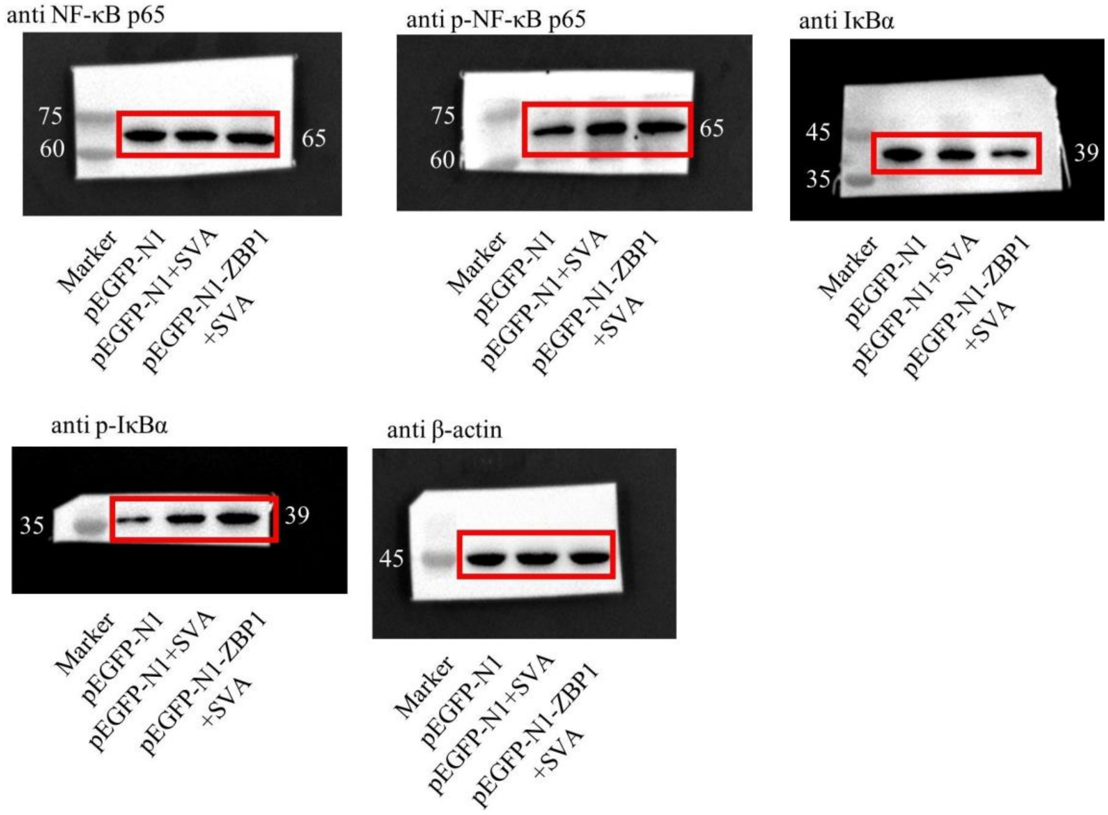

The third biological replicate

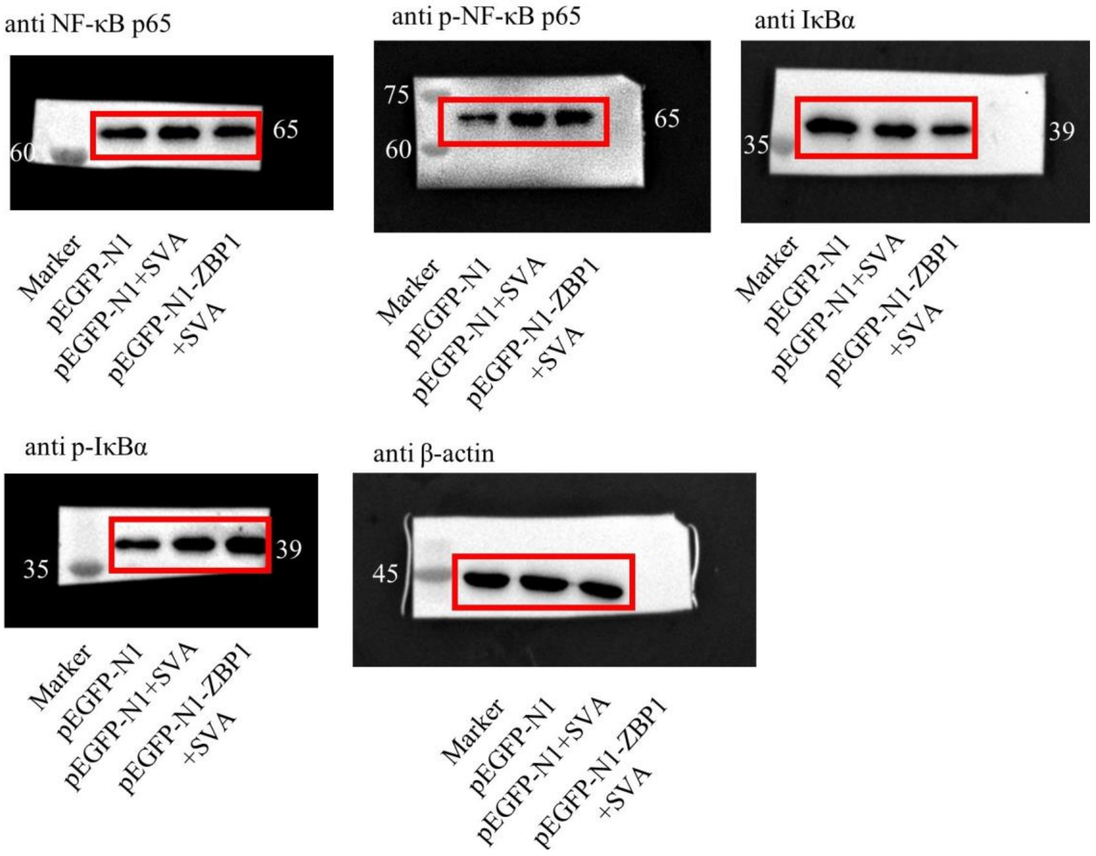

Raw image of western blot for NF-κB p65, p-NF-κB p65, IκBα, p-IκBα and β-actin (A).

C The first detect

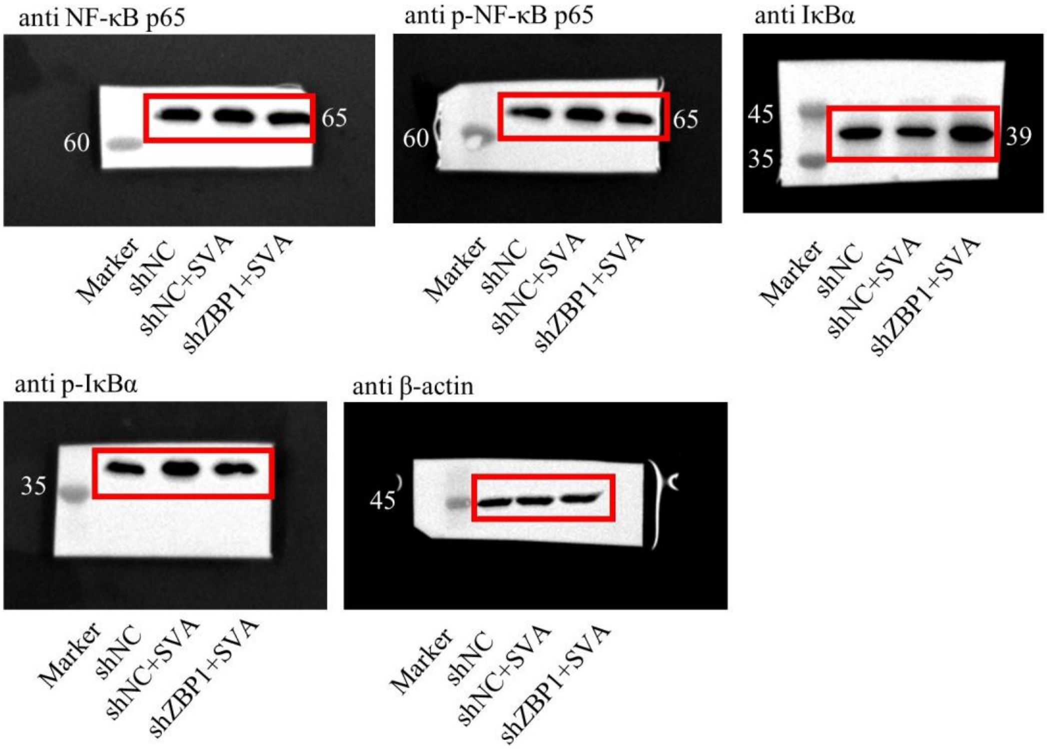

The second biological replicate

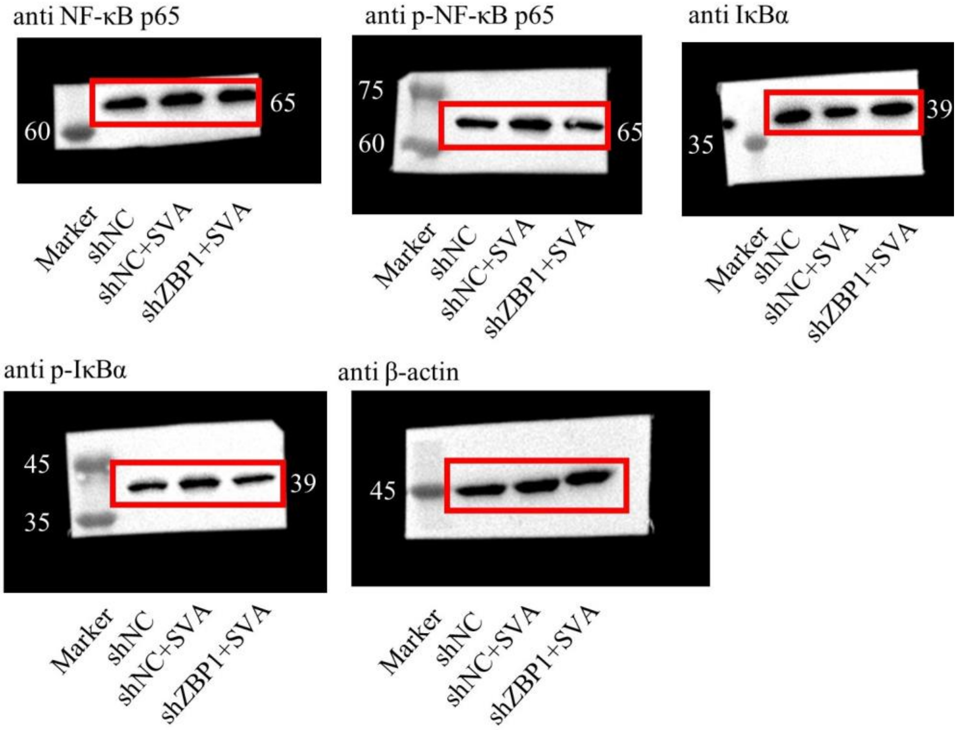

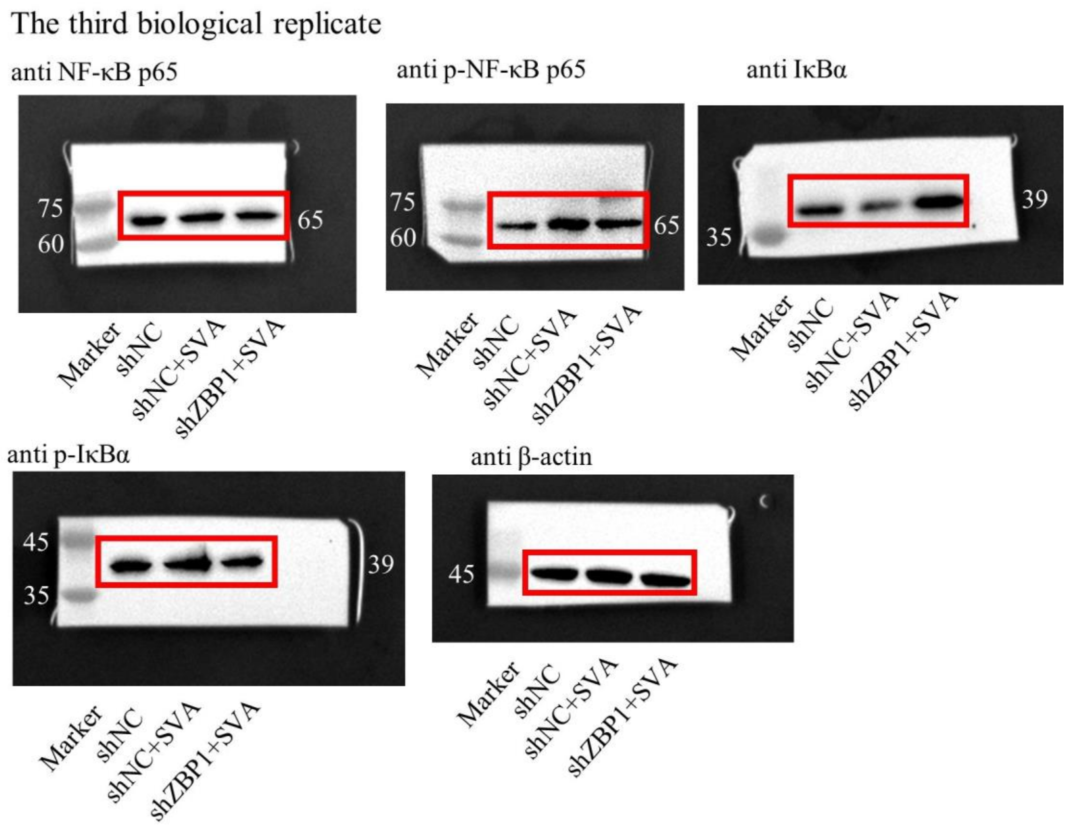

Raw image of western blot for NF-κB p65, p-NF-κB p65, IκBα, p-IκBα and β-actin (C).

Fig.5 ZBP1 activating NF-κB signaling pathway was verified by the specific inhibitor.

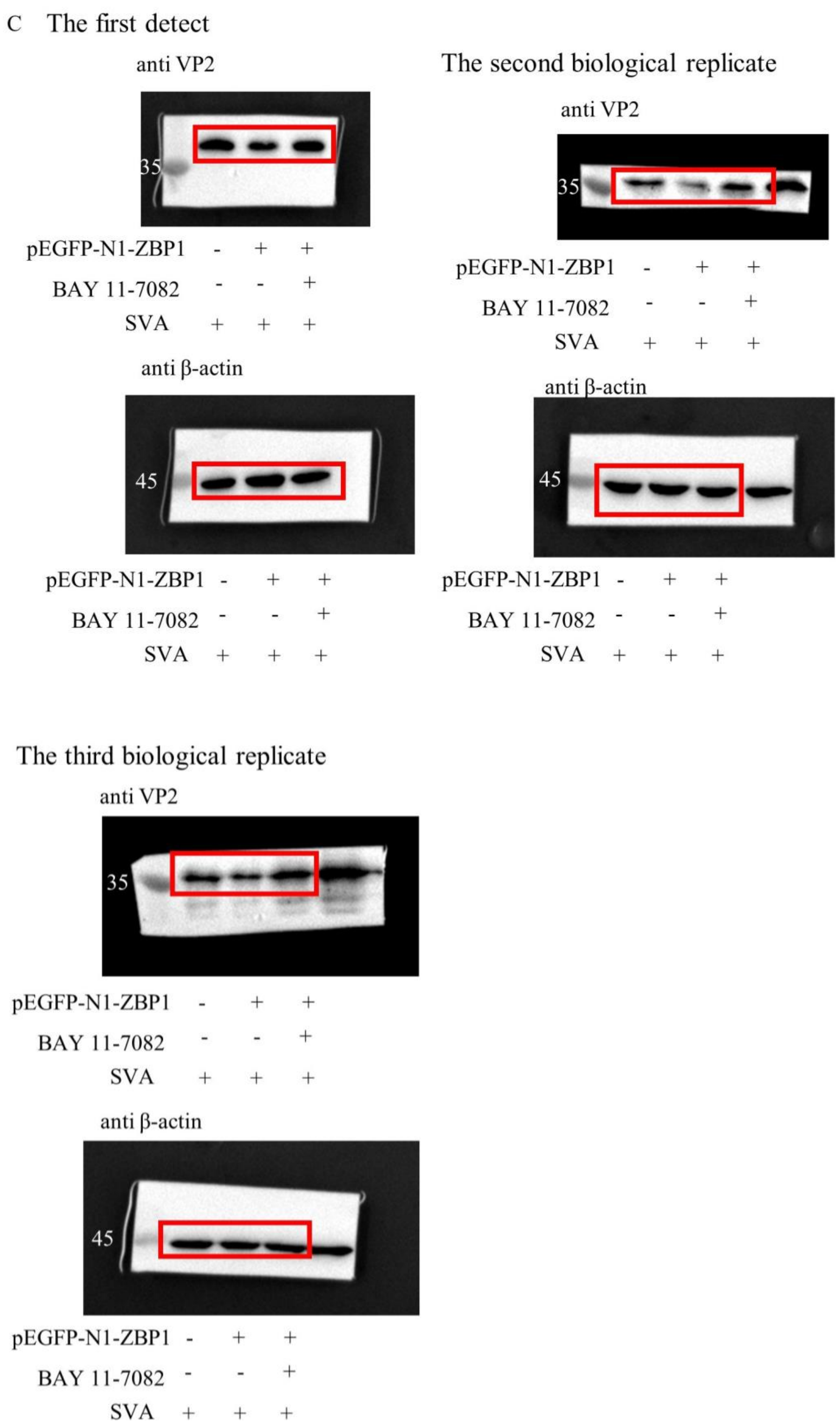

Raw image of western blot for VP2 and β-actin (C).

Supplementary Information
Additional file 1:
Supplementary Fig. 1

A

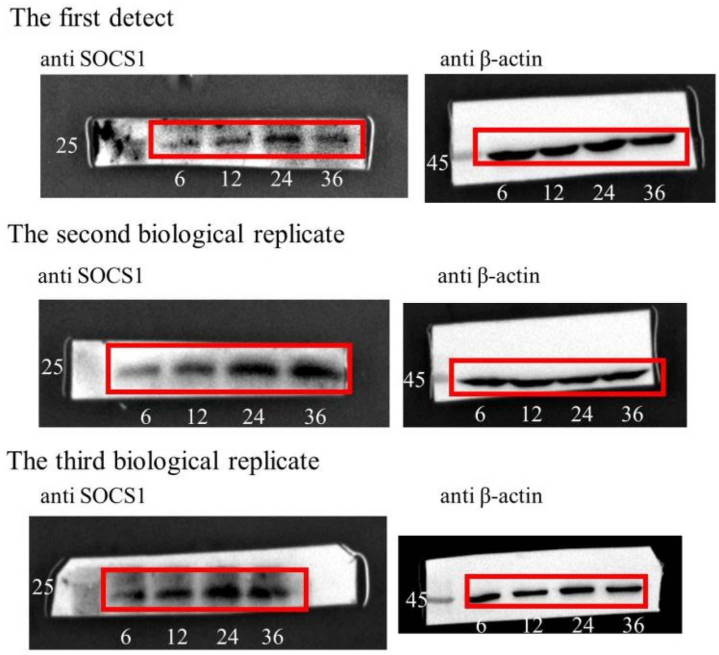

Raw image of western blot for SOCS1 in cells with overexpression of pEGFP-N1 (A).

B

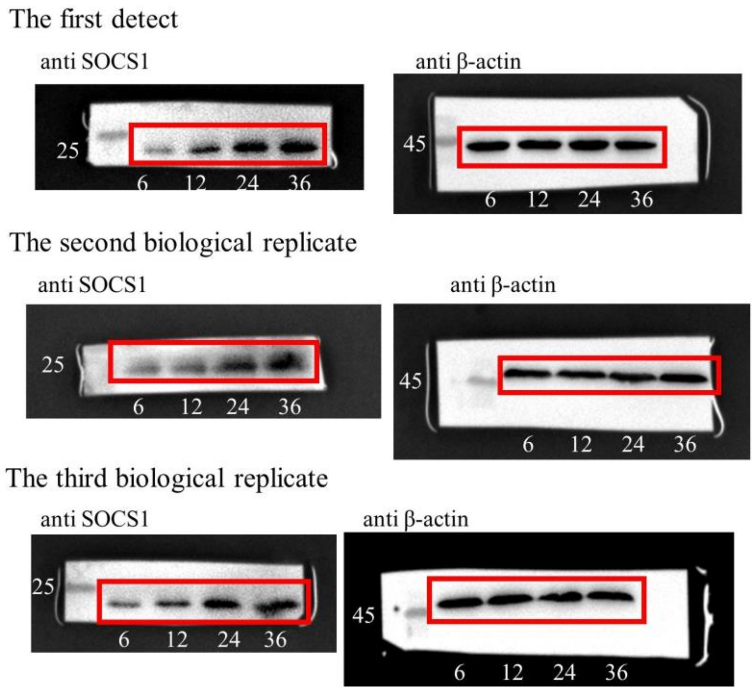

Raw image of western blot for SOCS1 in cells with overexpression of pEGFP-N1-ZBP1 (A).

Additional file 2:
Supplementary Fig. 2

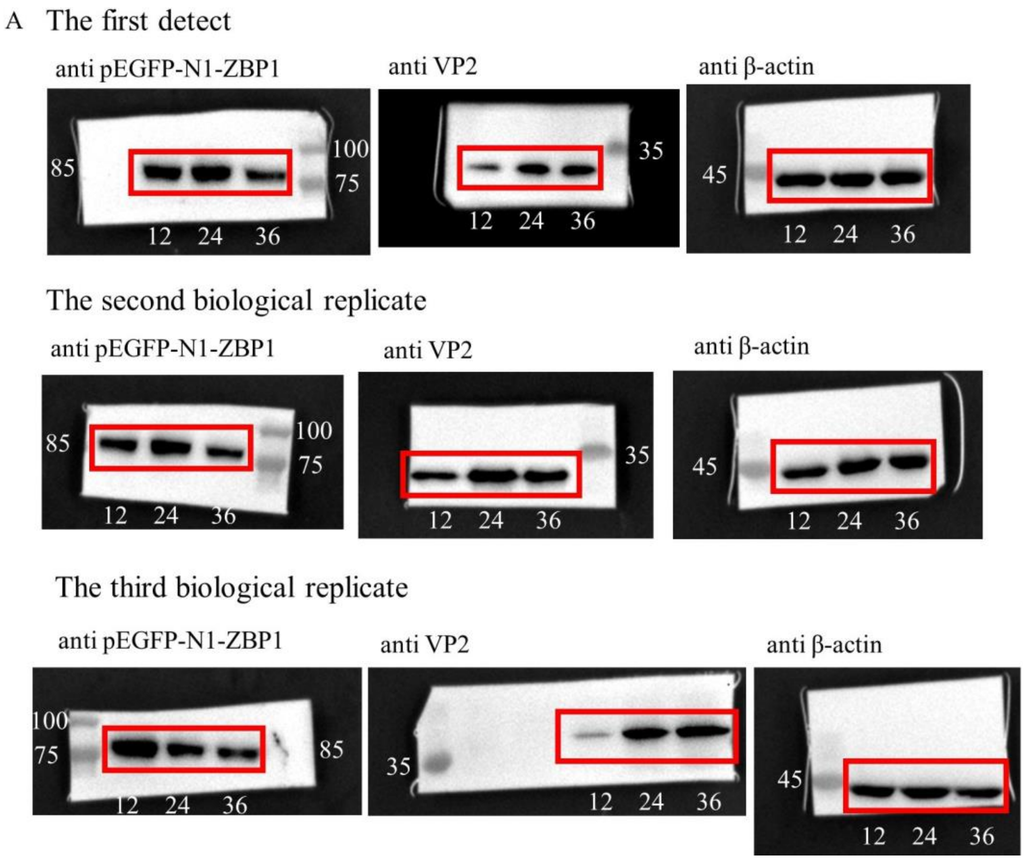

Raw image of western blot for pEGFP-N1-ZBP1, SVA VP2, and β-actin in cells with overexpression of pEGFP-N1-ZBP1 (A).

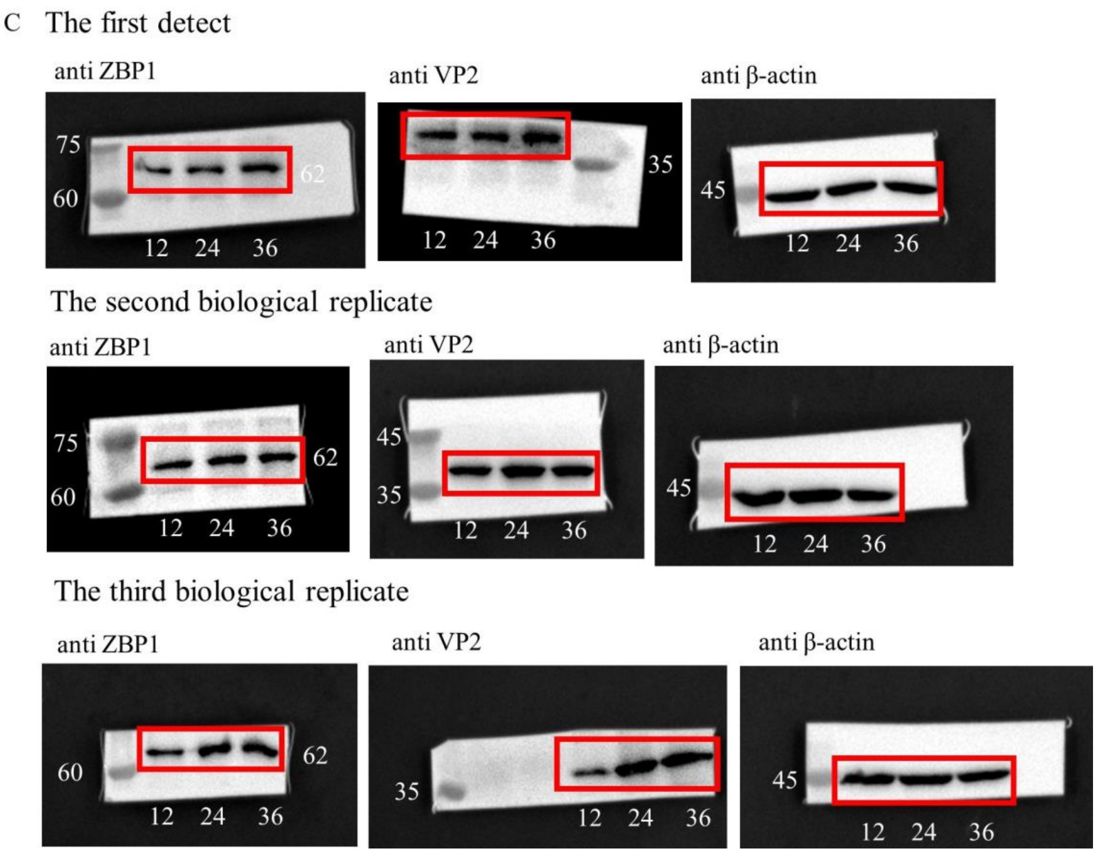

Raw image of western blot for pEGFP-N1-ZBP1, SVA VP2, and β-actin in cells with interference expression of ZBP1 (C).
